# How can modeling responsibly inform decision-making in malaria?

**DOI:** 10.1371/journal.pbio.3002991

**Published:** 2025-01-15

**Authors:** Jaline Gerardin, Melissa A. Penny

**Affiliations:** 1 Department of Preventive Medicine and Institute for Global Health, Northwestern University Feinberg School of Medicine, Chicago, Illinois, United States of America; 2 Applied Health Analytics for Delivery and Innovation, Chicago, Illinois, United States of America; 3 The Kids Research Institute Australia, Perth, Australia; 4 Centre for Child Health Research, The University of Western Australia, Perth, Australia

## Abstract

When models are used to inform decision-making, both their strengths and limitations must be considered. Using malaria as an example, this Perspective explains how and why models are limited and offers guidance for ensuring a model is well-suited for its intended purpose.

## Teaser

When models are used to inform decision-making, both their strengths and limitations must be considered. Using malaria as an example, we explain how and why models are limited and offer guidance for ensuring a model is well-suited for its intended purpose.

Mathematical models are sets of simplifying assumptions, based on current knowledge, about how the world works, followed to their logical (mathematical) conclusion. Different models make different sets of assumptions and are calibrated to different data sets. Models have been applied to problems in malaria for many years [1]. A century ago, models provided the theoretical basis for vector control, explaining why shortening the mosquito life span is one of the strongest methods for reducing transmission [2]. As our knowledge of the malaria system has grown, so has our ability to develop mechanistic transmission models—mathematical models that distill key disease dynamics and encode the processes of malaria infection and transmission into mathematical equations. These models are useful for simulation studies that explore processes for which there is no observed data and test possible scenarios.

Two decades ago, proponents argued that the role of models in evidence-informed decision-making for malaria should be to identify knowledge gaps and approximately compare alternative intervention strategies [3]. These remain the core strengths of mathematical models. However, since then, computational power has massively increased; malaria has received renewed focus at the global level and more funding; and malaria epidemiology and the intervention landscape have increased in complexity. The ambition for modeling has exploded, both in terms of what modelers are trying to accomplish and the expectations coming from decision-makers that models be capable of understanding and predicting ever more complex real-world effectiveness over time.

Models continue to be used to understand disease dynamics and their influencing factors, such as the evolution of resistance. However, models are now, implicitly or explicitly, also tasked with supporting decision-makers by providing quantitatively accurate predictions of lives saved or cases averted by certain interventions. During research and development (R&D) of novel malaria interventions, for example, transmission models are used to understand the potential impact of new tools before large-scale trials. Models are helping estimate the efficacy and duration requirements of new therapeutics [4], vaccines, and gene-drive mosquitoes [5]. Model predictions can also support decisions of where to target R&D resources or which delivery strategies to prioritize for deployment.

**Fig 1:**
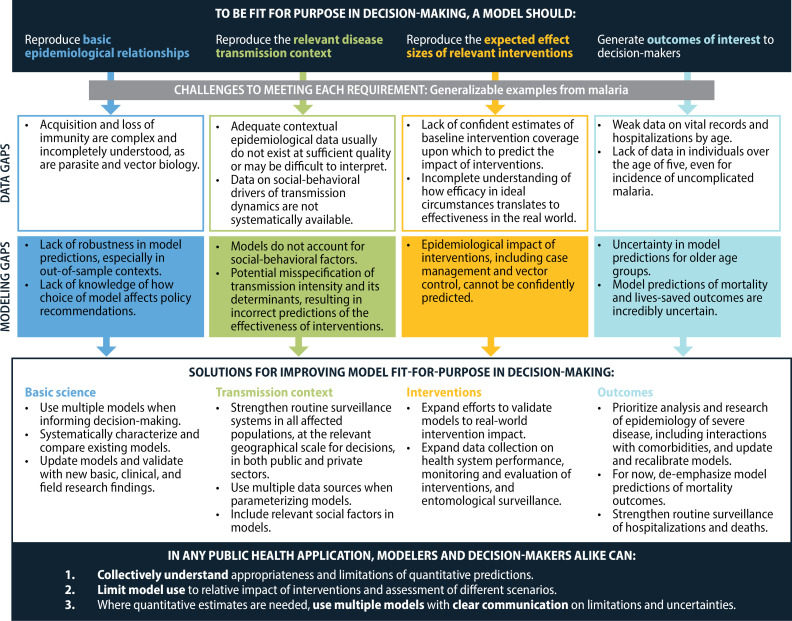
Ensuring a model is fit for purpose in decision-making. Any model should demonstrate that it is fit for purpose before it is used to inform decision-making. In malaria, key gaps persist that limit the ability of models to provide quantitatively accurate predictions of scenario outcomes. Solutions include the use of multiple models, continuous model improvement, expanded data collection, and transparency on model limitations.

Model outputs are also increasingly used as additional evidence when decision-makers set national intervention strategies or set global policy. Unfortunately, it is often forgotten that models must extrapolate to these desired output metrics with limited data, especially on mortality. Furthermore, it is not always evident that end users understand whether a model is fit for purpose (Fig 1), nor that modelers always adequately communicate model limitations in their haste to be part of decision-making. Decision-makers seldom request proof of rigor, trusting that modelers have validated their models and tested their conclusions. Some modelers assume decision-makers are not interested in or cannot understand the models’ inner workings, and gloss over critical limitations in pursuit of maximal application of the models. Recent rising demand for modeling has led several modeling groups to apply models as black box algorithms, and thus they do not themselves fully understand the models they use. Despite over a century of malaria model development and application, these disconnects are leading to potential misuse of models in decision-making. As a worst-case outcome, potentially wrong health policies are put in place, and eventually modelers are blamed for the consequences of misuse, with models being scapegoated as decision-makers try to evade accountability. However, when used well, models have tremendous value to rigorously inform our thinking about the future.

At the national level, intervention policies are set by local decision-makers under the leadership of national malaria programs. Modeling can be used to predict the impact of combinations of possible interventions and to understand the impact of past interventions [6]. However, data gaps regarding each country’s malaria context, and the exclusion of social factors by the models [7], mean that model results must have substantial uncertainty, even if the core model captures the natural history of malaria well.

At the global level, policymakers and funders such as the World Health Organization (WHO) and the Global Fund to Fight AIDS, Tuberculosis, and Malaria use models to forecast trends, estimate needs, assess gaps, set realistic targets, and understand the cost-effectiveness of interventions [8]. Outputs of global models are not intended (nor appropriate) to be applied to individual countries, even if they are sometimes supplied at the country level. Global-level modeling cannot capture all the necessary contextual information of specific countries, especially if country data and country expertise to inform key assumptions, the feasibility of intervention combinations, and priority outcomes were absent during model building.

The gaps summarized in Fig 1, which are not unique to malaria, necessitate that model results cannot be viewed as single quantitative predictions of the future—neither for malaria nor for other diseases. Therefore, overly quantitative applications of model outputs, such as using models to drive optimization algorithms for intervention planning to obtain a given health outcome, are inappropriate. While model outputs can give a useful idea of whether an intervention would have a large or small impact, they are not accurate enough to select between intervention combinations unless the difference in impact is large and robust to uncertainty in contextual factors.

Despite data challenges, models that are well-informed by local expertise remain incredibly useful when comparing the relative impact of different potential interventions and qualitatively assessing different strategies. This approach, where candidate intervention scenarios are generated through review and analysis of local data, and modeling is then used to estimate their relative impact, has been successfully used in several countries to prioritize intervention strategies [9].

Even when models are calibrated to the same reference data with comparable goodness of fit, they might not behave similarly in out-of-sample contexts due to differences in structural assumptions [10]. In other applications, such as forecasts of climate change and COVID-19 trends, decision-makers consider multiple models as an ensemble or with all results presented. Using multiple models from many teams provides more robust predictions and representation across multiple reasonable sets of assumptions, while still preserving the different uncertainties within and across models [11]. Unfortunately, multiple models are not sufficiently used when informing malaria decision-making (this includes work done by the authors). When making the investment case for malaria, the Global Fund has often relied on one mathematical model. Although not always achieved [12], WHO’s best practice is to request multiple models to provide evidence for impact and cost-effectiveness estimates [13,14]. GAVI, the Vaccine Alliance, uses three malaria models to assess the potential impact of various malaria vaccine rollout scenarios. At the national level, we are unaware if multiple models have ever been used during intervention planning.

Outside of a few consensus modeling exercises, it is not known whether different malaria models yield similar predictions of the impact of interventions. Systematically aligning and testing complex models requires substantial time commitments from experts in each model. However, continuing to avoid systematic model comparison does a disservice to the end users of model outputs, who risk receiving different answers to their questions depending on their choice of model.

Building trust and encouraging the uptake of useful modeling requires everyone to be honest about the limitations of each model. Otherwise, the overapplication (or worse, intentional or unintentional misuse) of models risks discrediting the entire field. Modelers have an obligation not to oversell and to be part of the conversation rather than advertise that they have the “quantitative” solution. Equally, those considering modeling evidence should ensure they understand the limitations, do not cherry-pick results to advocate for a particular conclusion, are willing to pressure-test results, and support improving models. We must all work together to ensure ensemble modeling becomes a more frequent reality and to continuously develop and improve models to push the boundaries of what is possible.

No doubt the modeling field can and should change tremendously over the next 10 years. As new technologies and high-quality data become available, the scope of appropriate applications for modeling will expand. For today, we should be ethical and rigorous in acknowledging what is ready for deployment in decision-making and what is still in development. Models are and have been useful in our fight to defeat malaria—let’s collectively ensure they remain so.
